# Cauterization of Cervix in Vomiting of Pregnancy

**Published:** 1878-10-20

**Authors:** John M. Boring

**Affiliations:** Atlanta, Ga.


### CAUTERIZATION OF CERVIX IN VOMITING OF PREGNANCY.

BY JOHN M. BORING, M.D., ATLANTA, GA.

My attention was called to an article on "The Vomiting of Pregnancy and its Treatment" published in the "London Lancet" in which is detailed the history of quite a number of cases of vomiting of pregnancy and its treatment by Dr. Jones, of Chicago, aud Dr. Marion Sims, of New York. I was so favorably impressed by the history of the cases detailed by Drs. Jones and Sims, that 1 resolved to give the treatment suggested, and so successfully practised by them, a fair test in the first case that fell into my hands. I had but a few days to wait before an opportunity presented itself.

On June 20, I was consulted by a gentleman living a few miles in the country in relation to his wife, who, he stated, could retain nothing on her stomach. After making the inquiry, as to her general health, etc., usual in such cases, I decided that pregnancy existed, and was the cause of all the trouble. I prescribed for the lady, and instructed her husband to let me hear from her in a day or two; if not relieved. He came back and reported her no better. I then prescribed alterative doses of sacharated calomel; also Creosote Emulsion, with Sub. Nit. Bismuth. In a few days he returned and reported no improvement, and stated that she was decidedly worse, and that he wanted me to see her immediately. I saw her in the afternoon of the sixth day, from the time of my first prescription. She was truly in a precarious condition; she could not retain anything in the stomach, not even ice; she was emaciated, and her countenance denoted great anxiety and distress. I then ordered Oxalate of Cerium, gr. ii. every two hours. I was not prepared to carry out the suggestions of Drs. Jones and Sims at that visit, as I had no speculum with me, I visited her again on the next day. I found her in, truly, a deplorable, and, to all appearances, alarming condition. She looked haggard and worn out—in fact, was completely prostrated; the husband and friends seemed apprehensive that she would certainly die. I resolved then to try the application of the Nitrate of Silver to the Os Uteri. After the necessary preparations I introduced the speculum and found the Cervix very large and soft; the os uteri not ulcerated, but imflamed; opened sufficiently to receive the index finger with ease. I applied the solid nitrate of silver to the os uteri, passing the pencil or stick of caustic, some three-fourths of an inch through the os into the cervix; I, of course, used care in making the application, so as to produce the whitened appearance usual on the application of caustic. All other treatment was stopped. I did not see or hear from the lady for one week. When I called to see her at the end of one week I was agreeably surprised to find her up and attending to her domestic affairs. She informed me that the sick stomach and vomiting ceased in one day after the application of the caustic, and that her appetite improved from the time the sickness ceased. I made another application of the caustic to the os as at the first application producing similar results of a whitened appearance. I have not visited the lady since; but have heard from her repeatedly. There has been no return of the sick stomach; her health is excellent. She is now in her seventh month of pregnancy. At the time of the cauterization she was about three and a half months advanced in pregnancy. I would here remark that this is her second pregnancy. She is a lady of fine general health; never sick much in her life; was married twelve years before the birth of the first child—a fine healthy boy. This case is so analagous to one or two included in the number of cases reported by Drs. Jones and Sims, and the treatment so effectual and satisfactory to myself and patient—putting an end to such agonizing suffering as she was enduring—that I am induced to present it for publication. I hope that my professional brethren will avail themselves of the first opportunity to try this remedy, and report the results to the Profession as I am firmly persauded that in it we have found a reliable remedy for this hitherto intractable and distressing affection.

